# Editorial: Volume II: fibrotic tissue remodeling as a driver of disease pathogenesis

**DOI:** 10.3389/fmolb.2024.1356591

**Published:** 2024-01-16

**Authors:** Arkadeep Mitra, Sarika Saraswati, Trayambak Basak

**Affiliations:** ^1^ Department of Zoology, City College (Affiliated to University of Calcutta), Kolkata, India; ^2^ Department of Biological Sciences, Tennessee State University, Nashville, TN, United States; ^3^ School of Biosciences and Bioengineering, IIT-Mandi, Mandi, Himachal Pradesh, India

**Keywords:** collagen, fibrosis, ECM-extracellular matrix, mass-spectrometry, RNA seq, animal model

Organ failure occurs when the resident tissues are unable to meet their metabolic needs and fail to perform their designated function. Many pathophysiological conditions can cause organ failure. Among them, fibrosis is a major contributor as it significantly perturbs the elasticity of the cells and renders them inefficient (Lieber and Ward, 2013). Initially, the research regarding organ pathophysiology was more focused towards deciphering the pathways leading to the death of the participating cells. However, in the last two decades, research on fibrotic tissue remodelling gained significant attention as it started to unravel subtle changes within the organ microenvironment during disease progression, not just in end-stage organ failure.

Fibrosis is a slowly developing phenomenon that eventually causes tissue degeneration, leading to devastating consequences in organs like heart, kidney, lung and liver (Conte, 2021). It occurs due to excessive accumulation of fibrous connective tissue in the extracellular matrix (ECM) area of injured tissues resulting in a fibrotic scar. The basic components of this fibrotic scar are—a mixture of fibrotic cells and collagens, chiefly types I and III. If this fibrotic tissue accumulation occurs beyond a threshold level, eventually organ malfunction occurs (Davidson et al., 2020). Several pro-fibrotic factors and cytokines have been proposed to be the major mediators of fibrosis in various tissues (Al-Hatt et al., 2022). With the rapid advancement of techniques like ECM proteomics, several novel molecules are also being reported as causal factors in this process (Naba, 2023).

In this issue, the objective was to delineate minute nuances of renal and cardiac fibrosis and their potential therapeutic measures. In this issue, two reviews, two original research articles and one perspective article encompassed the different aspects of fibrosis. Wang et al. contributed with a comprehensive review on renal fibrosis. The review summarized how renal fibrosis is a common manifestation of any chronic kidney disease. Although this occurs as a self-repair process in response to kidney damage, it seriously affects the renal filtration function and has almost no specific treatments (Basile et al., 2012). Hence, exploring targeted therapeutic measures is the need of the hour. It has been reported by many researchers that Histone deacetylases (HDACs) are major causal players in promoting renal fibrosis through epigenetic modifications involving non-histone (Tong, 2002; Kumar and Brooks, 2023). In this review, the authors summarized the signaling pathways modulated by HDACs in renal fibrosis. Furthermore, they scribed the mode of action of various HDAC inhibitors (HDACi) in the anti-fibrotic process. This elucidates HDACi as a novel therapeutic tool to slow down the progression of renal fibrosis.

In another review, Sarohi et al. summarized the role of Extracellular matrix (ECM) in maintaining elasticity in cardiac tissues. Dysregulated ECM remodeling causes fibrosis in the heart leads to stiffness in the cardiac tissues and culminates in heart failure (Piek et al., 2016; Sarohi and Basak, 2023). In this review, the authors have summarized the role of different ECM components during the progression of cardiac fibrosis. Very interestingly, they stressed upon the importance of applying proteome profiling to understand the key changes occurring in the ECM during the progression of fibrosis. In a nutshell, the review highlighted how mass spectrometry-based next-gen proteomics studies can broaden the potential to identify causal players to combat cardiac fibrosis develop more targeted therapeutic regimen.

In a perspective article, Reese-Petersen et al. pointed out that ECM proteins contain signaling domains that once released from the parent molecule may trigger cellular responses. Among these molecules, a very prominent one is endotrophin—a type VI collagen-derived fragment, whose circulatory levels is being considered as a risk marker in heart failure with preserved ejection fraction (HFpEF) (Staunstrup et al., 2021; Chirinos et al., 2022). In this perspective, the authors showed how stimulation of human cardiac fibroblasts by endotrophin can upregulate the synthesis of collagen type I, the main interstitial collagen that accumulates in the myocardium during fibrogenesis. These data provide a possible mechanistic explanation for the relation between circulating endotrophin levels and the risk of outcome in HFpEF. A very interesting work from Nanda et al. utilized the power of next-generation-sequencing-based methodology to portray the global transcriptomic changes occurring in the myocardium during cardiac fibrosis using a beta-adrenergic stimulation model. Their results highlighted the fact that ECM reorganization, cell-cell adhesion and structural genes were upregulated in their fibrosis model (Ricard-Blum et al., 2018). On the contrary, genes involved in cellular processes such as fatty acid oxidation, cardiac muscle contraction, etc. were downregulated.

Finally, in a study by Møller et al. the authors investigated the potential of endotrophin as a risk marker for complications to type 1 diabetes. In this study, Endotrophin was measured in serum and urine from 1,468 persons having type 1 diabetes. The outcomes included a composite kidney endpoint, first major adverse cardiovascular event (MACE), all-cause mortality, progression of albuminuria, incident heart failure, and sight-threatening diabetic eye disease. Cox proportional hazard models adjusted for conventional risk factors were applied to analyse the data. The analysis revealed that Urinary endotrophin was not associated with any outcome after adjustment, while Serum endotrophin level was found to be a risk marker for mortality and kidney complications in type 1 diabetes. Thus, serum endotrophin may serve as a biomarker of ECM remodeling, as it may identify persons with active pro-fibrotic processes at risk for complications in diabetes. This finding validates a previous study performed in a large and unselected cohort and may be important for designing novel intervention strategies to reduce fibrosis and consequent organ function loss.

The articles published in this issue have presented studies that revolved around the comprehensive proteomics, genomics, and epigenomic analyses of fibrotic diseases. Through mapping the enrichment of differentially expressed genes and proteins, the researchers have identified key fibrotic players including HDAC and endotrophin. Additionally, mass spectrometry-based next-gen proteomics studies identified extracellular matrix components crucial for cardiac fibrosis which was supported by cardiac fibrosis evaluation in a beta-adrenergic stimulation model. In summary, through comprehensive analyses of genes and proteins, the researchers have identified signalling pathways that may contribute to the fibrotic phenotype in multiple tissue injury models. Further evaluation of these key targets will be crucial in developing targeted therapies that can mitigate fibrotic diseases.
